# Between conservation and achievement: Hunters’ values and sustainability certification as a governance tool in recreational hunting

**DOI:** 10.1007/s13280-026-02412-z

**Published:** 2026-05-13

**Authors:** Olmo Linares, Mario Soliño, Juan Carranza, María Martínez-Jauregui

**Affiliations:** 1 Unidad de Investigación en Recursos Cinegéticos y Piscícolas (UIRCP), Universidad de Córdoba, Campus de Rabanales Colonia San José, Ctra. Nacional IV - A, Km 396, 14071 Córdoba, Spain; 2 Instituto de Investigaciones Marinas (IIM-CSIC), Rúa Eduardo Cabello 6, 36208 Vigo, Spain; 3 Instituto de Ciencias Forestales (ICIFOR, INIA‐CSIC), Ctra. de La Coruña km 7.5, 28040 Madrid, Spain

**Keywords:** Achievement motivation, Discrete choice experiment, Human–wildlife relations, Recreational hunting, Sustainability certification, Transformative governance

## Abstract

Understanding how resource users perceive and negotiate sustainability is essential for designing legitimate conservation governance. This study explores recreational hunters’ preferences for sustainability certification in Spain using a discrete choice experiment. Data were collected from 621 hunters representing both big game and small game modalities. Results show that hunters consistently prioritise ecological criteria—such as game population conservation, habitat management, and wild genetics—yet support for certification is conditional on maintaining satisfactory hunting outcomes. Pro-sustainability attitudes coexist with achievement-oriented motivations, with conservation valued when it sustains continuity, fairness, and authenticity of the hunting experience. These findings highlight how ecological and experiential rationalities can align rather than conflict. By revealing the conditions under which certification is perceived as acceptable, the study shows how sustainability standards can function as information-based governance tools that enhance legitimacy, trust, and adaptive management in recreational hunting systems.

**Supplementary Information:**

The online version contains supplementary material available at https://doi.org/10.1007/s13280-026-02412-z.

## Introduction

As natural systems face increasing threats from anthropogenic drivers of global change, understanding how societies use and govern natural resources has become a central scientific and policy priority (Díaz et al. 2019; Zoeller and Cumming 2024). Sustainable governance arrangements are essential to reconcile biodiversity conservation with human demands, particularly where extractive practices directly affect wildlife populations. Within this scenario, outdoor recreation constitutes a major interface between people and ecosystems, ranging from non-consumptive activities such as wildlife watching to consumptive practices such as fishing and hunting (Vaske and Reomer 2013). These activities illustrate how leisure and cultural practices can generate both ecological benefits and pressures, depending on how they are managed and perceived.

Recreational hunting, defined as the pursuit and killing of wild animals for leisure (Leader-Williams 2009), represents a particularly ambivalent case in outdoor recreation. It combines cultural heritage, rural economies, and social traditions, while at the same time being highly contested for its ecological, ethical, and legitimacy implications (Urbanik and Johnston 2017; Di Minin et al. 2021). This duality poses hunting at the center of broader debates about the sustainable and legitimate use of wildlife.

Across countries, institutions in a Northian sense (North 1990), i.e. formal rules and informal norms, regulate access to land and use of wildlife, defining who may hunt, under what conditions, and with what responsibilities (Rosser 2009). The allocation of wildlife rights and responsibilities varies internationally—from public trust regimes in North America to *res nullius* traditions in Europe, and mixed arrangements in Africa combining state, private, and community-based rights over wildlife (Chardonnet et al. 2002; Frost and Bond 2008; Smith 2011). This diversity reflects broader dynamics of access and control over natural resources (Ribot and Peluso 2003), but across contexts these arrangements share a common aim: to balance human use with biodiversity conservation, providing the governance context within which recreational hunting occurs. For the purposes of this study, hunting governance is understood as the set of formal rules, informal norms, and information- and market-based instruments that shape access to wildlife, management practices, and accountability in recreational hunting.

However, even within such frameworks, regulation has not prevented the persistence of unsustainable or ethically contentious management practices. This trend is particularly evident in market-oriented hunting systems across Southern Europe, where recreational hunting has undergone progressive intensification. Practices such as intensive management behind fences, predator eradication, supplementary feeding or the release of farm-reared animals are commonly used to artificially increase hunting bags (Apollonio et al. 2010; Mysterud 2010; Caro et al. 2014; Carpio et al. 2021). These interventions not only affect biodiversity but also erode social acceptance, threatening the “social licence” of hunting in contemporary societies (Hampton and Teh-White 2019; Meesters et al. 2021). Social research further indicates that such practices are also questioned by hunters, particularly in relation to their ecological consequences (Ghasemi and Kyle 2023).

In this context, recreational hunters often purchase access to hunting, equipment services, and hunting grounds, but much of the management process remains beyond hunters’ observation (Child and Wall 2009). This opacity creates an information asymmetry between hunters and estate managers (Park et al. 2022), turning sustainability into a *credence attribute* that cannot be directly verified (Darby and Karni 1973). When accountability mechanisms are weak or absent, responsible management cannot be effectively recognised or rewarded, allowing opportunistic or greenwashing strategies to emerge (Pascual-Fernández et al. 2018).

To address these informational weaknesses, new forms of information-based governance have emerged in natural resource management. They aim to enhance accountability by producing, validating, and communicating credible sustainability information (Mol 2006; Gupta 2008). Among these mechanisms, voluntary sustainability certification and eco-labelling represent the most institutionalised expressions of information-based governance, translating principles of transparency and credibility into auditable market instruments (Park et al. 2022). Originally developed in response to concerns over resource depletion and the limits of state regulation (Gulbrandsen 2005; Vogt 2019), this private governance mechanisms gained prominence across forestry, fisheries, aquaculture, and agriculture (Cashore et al. 2004; Foley 2019; Osmundsen et al. 2020; Dietz et al. 2021). Led by private actors yet often involving public oversight, these initiatives constitute non-state, market-driven systems that combine voluntary participation, transparency, and third-party verification to promote responsible management (Loconto and Busch 2010; Potts et al. 2014). Such instruments influence behaviour not through coercive regulation but through transparency, market signalling, and trust (Cashore 2017).

Comparable information-based governance approaches have gradually extended to the sustainable use of terrestrial wildlife (Fromentin et al. 2023). Although less developed than in other wildlife use industries, several initiatives illustrate the growing application of these governance tools to hunting and wildlife management (Child and Wall 2009). In the hunting sector, early and ongoing initiatives include criteria-and-indicators frameworks, voluntary certification schemes, collaborative management programmes, and monitoring networks aimed at standardising sustainability principles and performance indicators (Lexer et al. 2005; Fiorini et al. 2011; Keuling et al. 2011; Tack 2018; ENETWILD-consortium 2019; Mill et al. 2020; Pruitt et al. 2023; WE Label 2024). Building on these international experiences, a Spanish proposal has recently advanced the development of a third-party certification system for sustainable game management (Linares et al. 2024a). Given the scale and institutional complexity of recreational hunting in Spain (Apollonio et al. 2010; MAPAMA 2019), this context provides a suitable setting to explore how certification principles may be operationalised within wildlife governance.

Despite these advances, available evidence suggests that limited attention has been paid to how hunters themselves perceive and prioritise the sustainability criteria that underpin such certification systems (Park et al. 2022). Addressing this gap is crucial to ensure that governance tools are not only ecologically sound but also socially legitimate and responsive to the values of hunters, local communities, and other groups affected by recreational hunting. Accordingly, this study adopts a two-step design and pursues three objectives: (i) to identify and rank the sustainability criteria most valued by recreational hunters, informing the attribute set of a proposed certification scheme; (ii) to quantify preferences for these attributes using a discrete choice experiment, testing differences between big game and small game hunters; and (iii) to discuss implications for the design, legitimacy, and potential uptake of sustainability certification in recreational hunting.

## Materials and methods

### Study area

Spain hosts one of the most extensive and diverse hunting systems in Europe, covering nearly 85% of the national territory through more than 32 000 registered hunting estates and ranking as the second European country in terms of number of hunters (FACE 2007; MAPAMA 2019). The Spanish hunting community comprises over 725 000 licensed hunters (MAPAMA 2019), equivalent to 1.5% of the total population, though their distribution is uneven across regions and easingly male-biased (Gaspar et al. 2025).

This system has undergone profound transformations since the mid-twentieth century, driven by legal reforms, demographic shifts, and changing management models (Gaspar et al. 2025). The modern structure of hunting in Spain emerged from the *Ley de Caza* of 1970, which replaced the 1902 legislation and explicitly aimed to promote recreational hunting (Crespo-Guerrero 2017). This law, followed by the decentralisation of competencies to the 17 Autonomous Communities (AC) in 1989, marked the beginning of a plural and regionally governed framework (Martínez-Jauregui et al. 2011; Herruzo and Martínez-Jauregui 2013; Ruiz-Rodríguez et al. 2023). Each AC issues an annual hunting order that specifies open seasons, allowable methods, species quotas, and management restrictions, usually based on advisory committees including hunters, landowners, and environmental organisations (Piorno et al. 2020).

Under this regulatory architecture, landowners or managers must develop and periodically update Hunting Management Plans (HMPs) in accordance with regional laws. These plans report on game population trends, habitat conditions, and management practices such as predator control, restocking, or supplementary feeding (Ríos-Saldaña et al. 2018; Delibes-Mateos et al. 2021). Once approved, the plans grant hunting rights to the holder of the hunting ground for a period of four to five years, subject to annual reporting through the Annual Hunting Record (AHR). Aggregated data from these reports constitute the official national statistics on hunting yields (Martínez-Jauregui et al. 2011).

The diversity of game species reflects the country’s biogeographical complexity. Big game species include native ungulates such as red deer (*Cervus elaphus*), wild boar (*Sus scrofa*), roe deer (*Capreolus capreolus*), fallow deer (*Dama dama*), Iberian ibex (*Capra pyrenaica*), and chamois (*Rupicapra rupicapra*), alongside introduced species like mouflon (*Ovis orientalis musimon*) and Barbary sheep (*Ammotragus lervia*). Small game encompasses mammals such as the European rabbit (*Oryctolagus cuniculus*) and the Iberian hare, resident birds like the red-legged partridge (*Alectoris rufa*) and pheasant (*Phasianus colchicus*), and various migratory species including pigeons, thrushes, and waterfowl (MAPAMA 2019). Harvest statistics reveal a strong imbalance between both groups: while big game populations—especially red deer and wild boar—have expanded intensely, small game bags have experienced sharp declines, reshaping traditional hunting patterns (Herruzo and Martínez-Jauregui 2013).

Hunting modalities are equally diverse, ranging from *monterías* and stalking in big game to driven and walked-up shooting in small game (Macaulay et al. 2013; Caro et al. 2015; Linares et al. 2024b). Traditional practices, such as partridge decoy hunting (*reclamo*) and falconry, remain important cultural expressions that coexist with more modern forms of commercialised hunting (Vargas et al. 2006).

The historical expansion of big game hunting, particularly during the “Spanish hunting boom” of the 1970s (López Ontiveros and Verdugo 1991; Martínez-Garrido 2009), consolidated a commercialised model based on private estates and intensive management (Crespo-Guerrero 2017). Fenced estates are prevalent in the south and center, where productivist strategies—such as feeding, predator control, and restocking—are widespread, while northern areas maintain more extensive and collective forms of management (Ruiz-Rodríguez et al. 2023). This dual structure illustrates Spain’s complex socio-ecological mosaic, in which public, communal, and private hunting systems coexist under distinct management intensities and cultural traditions. These dynamics frame Spain as an archetypal case for analysing the social, ecological, and governance dimensions of recreational hunting in Southern Europe.

### Participants and data collection

Data for this study were collected through an online survey administered exclusively during July 2020 using the TickStat® platform. The online format proved particularly suitable for reaching hunters during the COVID-19 context of mid-2020, when restrictions on mobility and gatherings limited field-based surveys. The survey followed a non-probabilistic snowball sampling strategy (Parker et al. 2019). Initial contacts were established through hunting associations, professional networks, and personal contacts within the hunting sector, who were invited to distribute the questionnaire among other recreational hunters. This approach facilitated access to a geographically dispersed population but may have favoured respondents with higher levels of engagement or interest in management and sustainability issues (Naderifar et al. 2017). As with other snowball-based surveys, the sample cannot be considered statistically representative of all hunters in Spain. Consequently, the results should be interpreted as indicative of preference patterns among participating hunters rather than as population-level estimates. This strategy was considered appropriate given the absence of an updated national registry of hunters and the difficulty of accessing a dispersed population with heterogeneous affiliations and regional contexts. Following ethical research standards, all participants were informed about the purpose of the study, the voluntary nature of participation, the right to withdraw at any time, and the guarantee of anonymity and confidentiality of their responses.

A total of 621 hunters completed the questionnaire. Responses were obtained from hunters across the entire Spain, covering a broad range of autonomous communities and hunting modalities (see Table 1). Although the sampling method does not allow statistical inference to the total population, the sample represents the diversity of Spanish hunting practices in terms of geography, target species, and management systems. This limitation is consistent with previous studies on hunting in Spain, where the lack of detailed demographic data hinders assessing the representativeness of hunter samples (Martínez-Jauregui et al. 2011; Alba-Patiño et al. 2024).

**Table 1:** Socio-demographic characteristics of respondents (*n* = 621). Percentages are calculated over the hunter sample (*n* = 621). Autonomous community refers to the region from the location of respondents’ usual hunting estate

Characteristic	Category	*n*	%
Gender	Male	593	95.50
	Female	28	4.50
Age group (years)	18–25	165	26.60
	26–35	181	29.10
	36–45	158	25.40
	46–55	72	11.60
	56–65	36	5.80
	≥ 66	9	1.40
Type of hunting	Small game	380	61.20
	Big game	241	38.80
Autonomous community	Andalusia	169	27.20
	Extremadura	121	19.50
	Castilla-La Mancha	115	18.50
	Castile and Leon	84	13.50
	Valencian Community	28	4.50
	Aragon	23	3.70
	Galicia	16	2.60
	Community of Madrid	12	1.90
	Catalonia	11	1.80
	La Rioja	9	1.40
	Asturias	6	1.00
	Basque Autonomous Community	6	1.00
	Region of Murcia	6	1.00
	Navarre	5	0.80
	Balearic Islands	4	0.60
	Cantabria	4	0.60
	Canary Islands	2	0.30

### Questionnaire design

The questionnaire first gathered socio-demographic and contextual information about respondents and their hunting activity. Participants were asked whether they hunted big game, small game, or both; the characteristics of the area where they hunted most frequently (their usual hunting estate); and whether it was located in an open or fenced area. Additional questions addressed the region where the estate was located, the approximate distance from their residence, the motivations for hunting there, the number and type of hunting journeys undertaken during the previous season, the amount paid for those activities, and their level of satisfaction with the management of the estate (see Supplementary Information S1).

Subsequently, respondents were asked about their awareness of existing initiatives aimed at certifying sustainable hunting management and were introduced to the proposed Spanish certification system (Linares and Carranza 2019). The description explained that hunting estates may voluntarily submit their management practices to an independent audit in order to obtain certification for sustainable hunting management. To facilitate understanding, participants were provided with a concise explanation of the seven sustainability criteria forming the basis of this certification scheme (see Table 2).

**Table 2:** Seven sustainability criteria of game management used as attributes in the discrete choice experiment. Each criterion represents a dimension of sustainable hunting management included in the proposed Spanish third-party certification system (Linares et al. 2024a). The table presents the name of each criterion, its conceptual description, and the corresponding icon shown to respondents in the survey to facilitate understanding of the attributes during the choice experiment

Icon	Certification Criteria	Explanation of attributes
	C1.- Conservation of game population	Complying with this criterion implies: - Maintaining the population density, age and sex structure, and health status of hunted species at levels compatible with their conservation - Avoiding overexploitation and ensuring a harvest compatible with population renewal - Keeping hunting records and monitoring data on harvested individuals
	C2.- Promotion of wild and native genetics	Complying with this criterion implies: - Maintaining the pure, native genetics of hunted populations - Preserving wild genetic traits by avoiding management practices that alter natural selection processes - Not introducing farm-bred animals as a method to guarantee hunting opportunities
	C3.- Avoid commercial hunting of exotic species	Complying with this criterion implies: - Not introducing or releasing exotic species for game - Avoiding commercial hunting of non-native species such as sheep, mouflon, pheasants - Promoting only game species native to the area
	C4.- Maintain or improve non-game wildlife	Complying with this criterion implies: - Knowing which species inhabit the hunting estate and monitoring their presence - Caring for populations of wild species that are not hunted (e.g. small birds, raptors, lynx, reptiles)
	C5.- Manage and conserve habitats	Complying with this criterion implies: - Renewal of trees and woody plants - Avoidance of trampling and soil erosion - Preservation of hedges, boundaries, and vegetation patches - Promotion of appropriate phytosanitary product use - Agricultural work that respects the breeding periods of species
	C6.- Promote local employment and worker training	Complying with this criterion implies: - Providing training and professional development for gamekeepers and other workers - Generating local employment linked to hunting management
	C7.- Increase hunter satisfaction	Compliance with this criterion means that: - Hunter satisfaction is recorded at the end of the hunt - Hunters have mechanisms to complain if what was promised is not delivered
	Cost of certification	We chose an increase of 5%, 10%, 15%, and 20% of what the hunter paid for all their hunting journals in their usual hunting area during the last season. If they did not pay for the hunting days, they were shown the value of € 30, € 60, € 90, and € 120

Participants were then asked to evaluate the importance of each criterion for achieving sustainable hunting management using a ten-point scale (ranging from not important to very important and a “don’t know” option). Do not know responses (*n* = 364) were deleted from the calculation of these statistics. They were also asked whether they believed each criterion was adequately respected in their usual hunting estate. Finally, a discrete choice experiment (DCE) was presented to assess hunters’ preferences for certification programs.

### Certification attributes

The experiment was designed to estimate hunters’ preferences and willingness to pay (WTP) for the seven sustainability attributes corresponding to the certification criteria described previously. Each attribute represented one of the dimensions of sustainable game management, while an additional cost attribute captured the monetary trade-off associated with certification. The attributes used in this study correspond to seven sustainability criteria designed to define the key dimensions of sustainable game management for certification purposes (see Linares et al. 2024a). The criteria were consolidated involving hunting representatives, policymakers, conservation scientists, and game managers, using a Delphi-based approach to build consensus among participants with diverse perspectives on game management sustainability. The final set was designed to capture the ecological, social, and governance dimensions that are essential for defining sustainability in recreational hunting systems.

The seven criteria identified are presented in Table 2. They encompass (i) game population status, referring to the monitoring and conservation of species abundance and structure; (ii) genetic integrity, ensuring the avoidance of artificial practices such as indiscriminate translocations or hybridisation; (iii) avoid exotic species, preventing the release or promotion of non-native fauna in hunting estates; (iv) maintain or improve non-game wildlife status; (v) habitat stewardship, reflecting active efforts to maintain or restore ecological functions and habitats that support wildlife; (vi) local socioeconomic benefits, including contributions to rural employment and income; and (vii) hunter satisfaction, as a measure of the experiential quality and legitimacy of hunting practices. In addition, an eighth attribute represented the cost of certification, expressed as a percentage increase in the total amount the hunter currently pays for hunting days in their usual estate. Following previous literature (Soliño et al. 2017), four cost levels were used: 5%, 10%, 15%, and 20%. For hunters who did not pay directly for hunting, a cost vector (€30, €60, €90, and €120) was used, corresponding to the approximate result of applying the same percentage range to the average expenditures reported in a pilot survey. The cost attribute thus represents the additional amount hunters would need to contribute if the management of their usual hunting estate was certified.

These seven certification criteria were introduced to respondents in the survey as the basis for evaluating alternative certification scenarios. For each attribute, levels were defined to represent realistic management outcomes and trade-offs between more and less sustainable practices. Together, the attributes were intended to represent a comprehensive framework of sustainability in game management, moving beyond purely ecological indicators to also include ethical and social dimensions that influence the legitimacy and acceptability of hunting in Spain.

### Choice experiment

Discrete choice experiments (DCEs) are a stated-preference method widely used in environmental and resource economics to elicit preferences for non-marketed goods and services. Incorporating both use and non-use values (Lancaster 1966; Johnston et al. 2017), DCEs were also broadly applied in research on outdoor recreation (Pröbstl-Haider et al. 2020). By asking participants to evaluate trade-offs among management attributes, DCEs not only quantify individual utility but also reveal shared normative thresholds—what resource users collectively consider acceptable or desirable practices (Castillo-Eguskitza et al. 2019). In the context of hunting governance, this approach offers a means of exploring the intersection between individual motivations and collective arrangements. In certification schemes, these collective arrangements take the form of shared rules and norms that become codified as standards, as hunters’ choices express both their personal preferences and their willingness to align with those shared rules (Fischer et al. 2015). Before starting the experiment, respondents were given detailed explanations of the certification concept, the seven sustainability criteria, and the meaning of each attribute and level. They were informed that certification would entail an additional cost for landowners, which could be transferred to hunters, but that it would not directly alter hunting outcomes. Participants were reminded of their budget constraints and told that choosing the non-certification option was entirely valid and lawful choice.

To capture how hunters evaluate certification attributes relative to their current hunting experience, two alternative representations of the *status quo* (SQ) were considered: a provided SQ and a perceived SQ. The provided SQ refers to the baseline scenario described in the choice experiment, in which none of the sustainability criteria were fulfilled. By contrast, the perceived SQ reflects respondents’ own assessment of whether these criteria were already fulfilled in their usual hunting areas. Prior to the choice tasks, hunters indicated whether each sustainability criterion was respected in the estate where they most frequently hunt. These responses were used to construct an individualised perceived SQ. Following Domínguez-Torreiro and Soliño (2011), separate Random Parameters Logit models were estimated for big game and small game hunters under both SQ specifications. This approach allows examination of whether preferences for certification differ depending on whether sustainability is framed as an external improvement or as recognition of existing management practices.

**Fig. 1:**
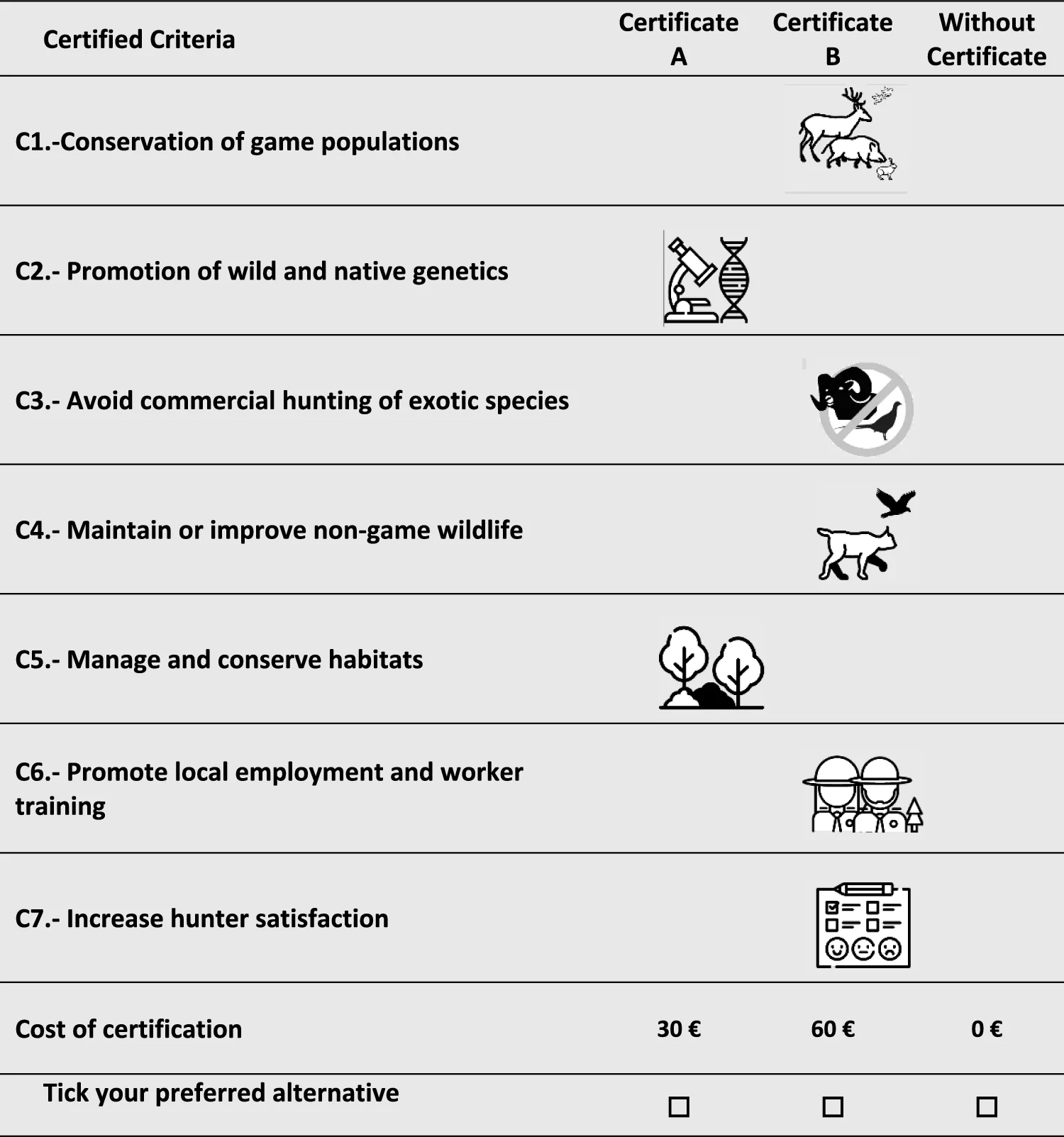
Example of a discrete choice card used in the survey. Certificate A shows that compliance with Criteria 2 and 5 (promotion of native species and habitat management) is certified and that this results in an increase in the cost of hunting journeys on the respondent’s usual hunting ground of €30. Certificate B shows that compliance with Criteria 1, 3, 4, 6, and 7 is certified (game population conservation, management of exotic species and non-game fauna, promotion of local employment, and increment of hunter satisfaction), resulting in a cost increase of €60. The third option (“Without certificate”) represents the status quo with no cost (€0)

A total of 512 scenarios were possible. The experimental design followed a D-optimality criteria and was conducted using the Ngene® software (ChoiceMetrics 2012). The design ensured a manageable number of choice cards (see Annex S1) with an associate D optimality of 84.59%. Each choice card contained two certification alternatives (Certificates A and B) and a status quo or opt-out alternative (“Without certificate”). They were randomly showed to each participant. Each alternative combined different sets of certified criteria and a corresponding cost increase (Fig. 1). Respondents were asked to select their preferred alternative on each choice card.

Choice data were analysed using a Random Parameters Logit (RPL) model (Train 2009), which accounts for unobserved heterogeneity in preferences by allowing coefficients to vary randomly across individuals. RPL models are widely employed in environmental valuation studies because they capture differences in preferences that are not explained by observable characteristics (Sagebiel 2017; Cerdá et al. 2024). The model specification assumed normal distributions for random parameters and included an alternative-specific constant (ASC) defined as a dummy variable capturing the average preference for certified alternatives relative to the *status quo* (e.g. Boxall et al. 2009; Domínguez-Torreiro and Soliño 2011; Sagebiel 2017). The cost attribute was specified as a fixed parameter, given its limited explanatory power in preliminary model diagnostics. We have tested log-normal, triangular, and normal distributions of the cost parameter.

## Results

The sample included 241 big game hunters and 380 small game hunters. They were predominantly male (95%) and ranged in age from 18 to 88 years (see Table 1). The usual hunting grounds (defined as those most frequently visited in the last hunting season) selected for the questionnaire were located in all regions (Autonomous Communities) of Spain, 50% were collectively managed hunting grounds (usually managed by local hunters), and the other 50% were private hunting grounds. Among the hunting grounds reported by respondents, 12% corresponded to perimeter fenced hunting grounds. A total of 7754 big game hunting days were reported (30% hunts with hounds, 30% wild boar night waiting, 23% trophy stalking, etc.), which corresponds to an average of 32 hunting days per season and hunter. 22 big game hunters did not pay (invited or self-consumption) and the rest paid very variable prices (average € 1724, Std.Dev. = 3408). For small game, respondents reported a total of 12 726 hunting days, which corresponds to an average of 33 hunting days per season and hunter, of which 40% were stalking hunts and 25% were driven hunts. Only 10 small game hunters reported no payment for hunting, the rest paid an average of €446 with a Std.Dev = 573. In both cases, the small subset of respondents reported not paying directly for access to hunting areas, typically due to membership in hunting societies or access to communal or family-owned estates. These respondents were retained in the analysis, as they still face opportunity costs and management conditions relevant to the choice scenarios.

Table 3 shows that, based on respondents’ direct self-reported assessments collected prior to the choice experiment, for both big game and small game respondents, all of the criteria considered ranked above 7 in importance, with *Conservation of game population*, *Habitat management*, *Promotion of wild and native game species* and *Maintain or improve non-game wildlife* being the most important (greater than or equal to 8.5 out of 10). The table also shows that the majority of respondents perceive that the criteria are met on their usual hunting grounds (where the choice experiment exercise is carried out), except for the criterion of Increasing hunter satisfaction for big game (48.1% of respondents perceive that the criterion is met), and Habitat management (46.6%) and Increasing hunter satisfaction (43.2%) for small game.

**Table 3:** Rating of the importance of the criterion for achieving sustainable wildlife management (10-point scale)

	Big game (*N* = 241)		Small game (*N* = 380)	
	Average rating (Std. Dev.)	% of respondents confirming that the criteria are already accomplished	Average rating (Std. Dev.)	% of respondents confirming that the criteria are already accomplished
*C1: Conservation of game population*	9.1 (1.36)	68.9	9.1 (1.59)	63.4
*C2: Promotion of wild and native genetics*	8.9 (1.64)	80.1	8.6 (2.08)	67.6
*C3: Avoid commercial hunting of exotic species*	7.2 (3.13)	75.1	7.4 (2.93)	76.8
*C4: Maintain or improve non-game wildlife*	8.6 (1.98)	78.8	8.5 (2.26)	71.6
*C5: Habitat management*	8.8 (1.88)	66.4	8.9 (2.01)	46.6
*C6: Promotion of local employment and worker training*	8.2 (2.27)	60.2	8.0 (2.43)	52.6
*C7: Increase hunter satisfaction*	7.5 (2.82)	48.1	7.4 (2.70)	43.2

**Fig. 2:**
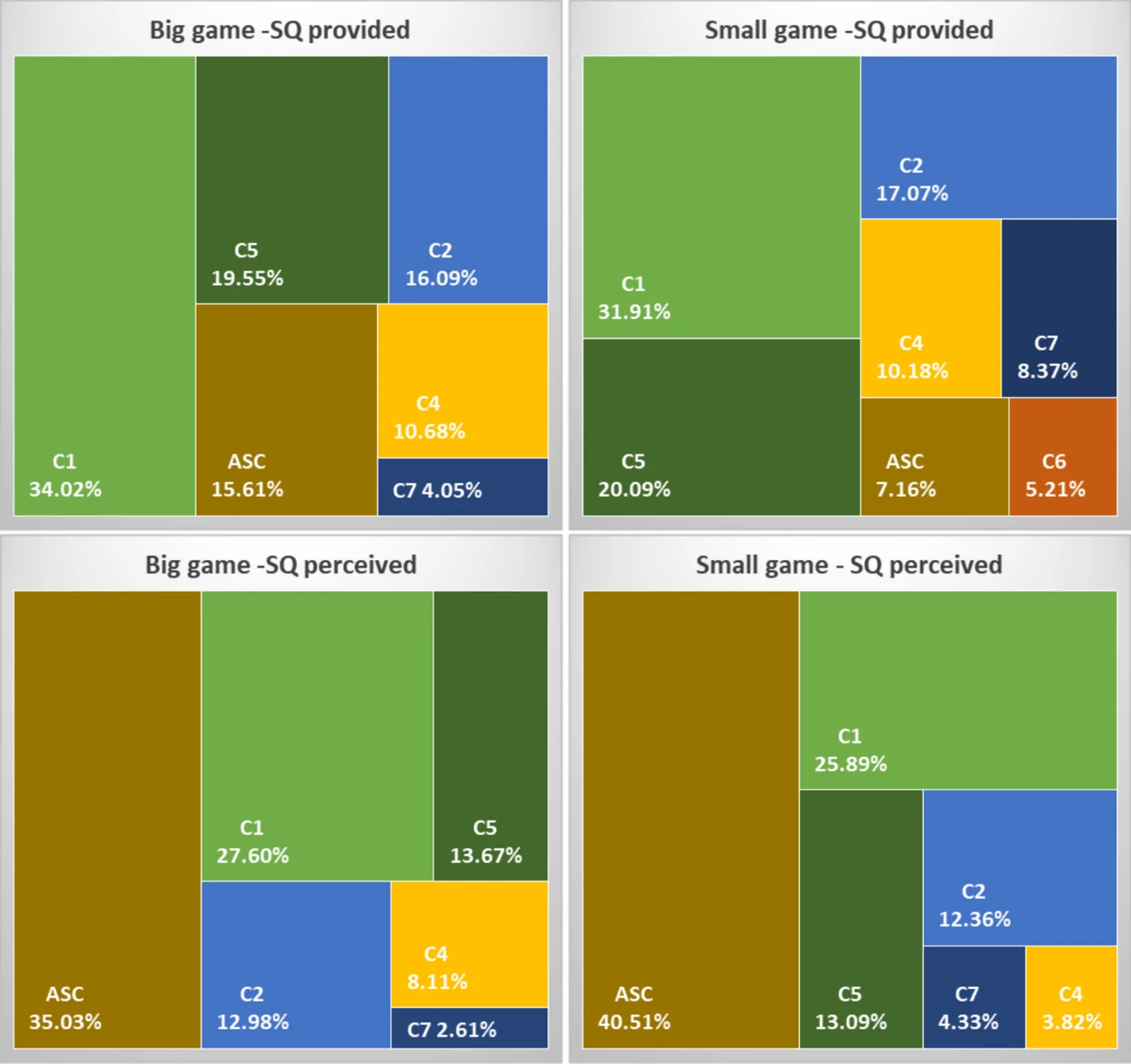
Relative weight of the significant certification criteria of the Random Parameters Logit Models analysed (C1: *Conservation of game population*; C2: *Promotion of wild and native genetics*; C3: *Avoid commercial hunting of exotic species*; C4: *Maintain or improve non-game wildlife*; C5*: Habitat management*; C6: *Promotion of local employment and worker training*; C7: *Increasing hunter satisfaction*). Results are presented according to status quo conditions and hunting modalities: (**a**) Big game—SQ provided; (**b**) Small game—SQ provided; (**c**) Big game—SQ perceived; (d) Small game—SQ perceived

The results of the Random Parameter Logit models are shown in Table 4. Using the two alternative status quo specifications defined above (SQ provided and SQ perceived), our results generally show that the preferences for the criteria to be certified are maintained in the big game and small game models: *Conservation of game population*, *Habitat management*, *Promotion of wild and native genetics*, *Maintain or improve non-game wildlife*, and *Increasing hunter satisfaction* in order of preference (Table 4 and Fig. 2), except for the criteria *Increasing hunter satisfaction* and *non-game wildlife*, which are reversed in the small game model using the SQ perceived, and *Promotion of local employment and worker training*, which appears as significant in the small game model when SQ provided is considered. This is consistent with the results on the importance of each criterion in the previous questions (see Table 3). The statistical significance and absolute values of the estimates for the monetary attribute suggest that respondents are not affected by an income effect. Thus, we cannot compute the willingness to pay for each of the attributes of the label certifying programs. In both model specifications, the alternative-specific constant (ASC) captures the average utility associated with choosing a certified hunting estate relative to the SQ, after accounting for the effects of the individual sustainability attributes. Differences in the sign and magnitude of the ASC between the provided and perceived SQ models therefore indicate how hunters’ baseline valuation of certification depends on whether certification is framed as an improvement over, or a validation of, current management practices. Then, the sign of the alternative-specific constant (ASC) changes, suggesting a shift from unwillingness to pay for a certification label to willingness to pay for it.

**Table 4:** Coefficient (Standard Error) of the random parameters logit models (Significance at 1% level:***; 5%;**; and 10%:*)

	SQ provided		SQ perceived	
	Big game	Small game	Big game	Small game
	Coefficient (Standard Error)	Coefficient (Standard Error)	Coefficient (Standard Error)	Coefficient (Standard Error)
*C1: Conservation of game population*	1.497*** (0.142)	1.426*** (0.135)	1.490*** (0.127)	1.131*** (0.121)
*C2: Promotion of wild and native genetics*	0.708*** (0.112)	0.763*** (0.102)	0.701*** (0.098)	0.540*** (0.086)
*C3: Avoid commercial hunting of exotic species*	0.034 (0.105)	0.067 (0.093)	0.983 (0.086)	− 0.025 (0.077)
*C4: Maintain or improve non-game wildlife*	0.470*** (0.098)	0.455*** (0.092)	0.438*** (0.084)	0.167** (0.081)
*C5: Habitat management*	0.860*** (0.104)	0.898*** (0.108)	0.738*** (0.085)	0.572*** (0.096)
*C6: Promotion of local employment and worker training*	0.076 (0.087)	0.233** (0.093)	0.010 (0.083)	0.127 (0.083)
*C7: Increasing hunter satisfaction*	0.178** (0.081)	0.374*** (0.080)	0.141* (0.076)	0.189*** (0.070)
***ASC***	− 0.687***(0.138)	− 0.320** (0.142)	1.891*** (0.100)	1.770*** (0.100)
COST	− 0.001***(< 0.001)	− 0.001 (< 0.001)	− 0.0005***(0.0002)	− 0.0007 (0.0007)
***Distributions of Random Parameters***				
*C1: Conservation of game population*	1.876*** (0.136)	1.856*** (0.135)	1.616*** (0.133)	1.662*** (0.126)
*C2: Promotion of wild and native genetics*	1.294*** (0.114)	1.173*** (0.104)	1.101*** (0.104)	1.038*** (0.110)
*C3: Avoid commercial hunting of exotic species*	1.244*** (0.115)	0.995*** (0.096)	1.017*** (0.110)	0.834*** (0.091)
*C4: Maintain or improve non-game wildlife*	1.005*** (0.105)	0.960*** (0.096)	0.882*** (0.985)	0.982*** (0.105)
*C5: Habitat management*	1.110*** (0.112)	1.250*** (0.103)	0.892*** (0.110)	1.282*** (0.104)
*C6: Promotion of local employment and worker training*	0.777*** (0.104)	0.995*** (0.102)	0.917*** (0.103)	1.043*** (0.103)
*C7: Increasing hunter satisfaction*	0.653*** (0.114)	0.747*** (0.101)	0.788*** (0.107)	0.685*** (0.096)
***McFadden Pseudo R2***	0.315	0.343	0.256	0.282

## Discussion

This study examined recreational hunters’ preferences for sustainability criteria included in a proposed third-party certification scheme for game management in Spain, using a discrete choice experiment. By comparing preferences across big game and small game hunters, and under alternative representations of the SQ (provided versus perceived), the analysis aimed to clarify how hunters value certification attributes and how certification is positioned relative to current hunting practices. The results provide insight into both the relative importance of specific sustainability criteria and the conditions under which certification is perceived as attractive or acceptable.

### SQ framing and interpretation of the ASC

A central finding concerns the alternative-specific constant (ASC), which captures hunters’ baseline valuation of certification relative to the SQ once the effects of individual sustainability attributes are accounted for. The sign and significance of the ASC differed systematically between the provided and perceived SQ models. When certification was evaluated against a standardised, non-certified baseline (provided SQ), the ASC was negative or non-significant, indicating reluctance to choose certification when framed as an external change to current practices. In contrast, when certification was evaluated relative to hunters’ own assessments of management conditions in their usual hunting estates (perceived SQ), the ASC became positive, suggesting greater acceptance when certification is framed as recognition of practices already in place.

This pattern highlights the importance of framing effects in shaping hunters’ responses to certification. Rather than reflecting unconditional support for or opposition to certification, baseline preferences depend on whether certification is perceived as an imposed requirement or as a mechanism that validates existing management efforts. Such conditional support aligns with previous work showing that hunters are more receptive to governance instruments perceived as fair, recognition-based, and compatible with their autonomy and experiential goals (Decker and Connelly 1989; Vaske and Roemer 2013). More broadly, these findings resonate with the logic of voluntary sustainability standards, which rely on credibility, transparency, and trust rather than coercive regulation to gain legitimacy among resource users (Blackman and Rivera 2011; Potts et al. 2014).

### Preferences for sustainability criteria

Beyond framing effects, hunters expressed clear and consistent preferences for several sustainability criteria. Across hunting modalities and model specifications, criteria related to the conservation of game populations (C1), habitat management (C5), and the promotion of wild and native genetics (C2) ranked among the most valued attributes. These preferences indicate a strong emphasis on ecological conditions that underpin both the continuity and the quality of hunting opportunities.

The high valuation of game population conservation reflects the importance hunters attach to maintaining population density, structure, and health compatible with long-term viability. For big game hunters, this preference is consistent with concerns about overabundance and its ecological consequences (Perea et al. 2014; Carpio et al. 2021). For small game hunters, it aligns with the need to preserve viable populations of wild origin to sustain harvests without depleting natural stocks (Díaz-Fernández et al. 2013). At the same time, these preferences also reveal a behavioural logic: conserving game populations contributes to hunting success and continuity, linking ecological concern with achievement-oriented motivations (Decker et al. 1980; Fulton and Manfredo 2004; Brunke and Hunt 2008). While these motivations often reinforce each other, they may diverge when sustainability constraints limit harvest opportunities (Williams 1989; Manning 2011).

Habitat management (C5) was similarly valued, although less than half of small game hunters perceived this criterion as adequately fulfilled. This perception gap suggests recognised scope for improving practices such as habitat restoration, soil conservation, and coordination with agricultural activities. Well-managed habitats not only support game populations but also enhance the experiential quality of hunting, reinforcing the link between ecological benefits and enjoyment (Adams and Steen 1997; Delibes-Mateos et al. 2014). These results echo broader European policy and societal trends emphasising biodiversity-friendly land management (Martínez-Jauregui et al. 2021) and point to certification as a potential instrument to incentivise habitat stewardship in hunting estates.

The promotion of wild and native genetics (C2) also ranked highly for both big and small game hunters, indicating shared disapproval of strongly artificial management practices such as indiscriminate restocking. However, the relatively low importance assigned to avoiding commercial hunting of exotic species (C3) reveals a trade-off within hunters’ preferences. While genetic integrity is valued as part of ecological authenticity and hunting identity, excluding exotic species may conflict with economic or recreational interests, particularly in commercial hunting systems (Caro et al. 2014; Carpio et al. 2017). This pattern reflects ongoing debates around “wildness” and ethical responsibility in contemporary wildlife management (von Essen and Allen 2020).

Preferences for the maintenance or improvement of non-game wildlife conservation (C4) were more context-dependent. Big game hunters tended to assign greater importance to this criterion than small game hunters, for whom non-game species, including predators, may be perceived as competitors. This divergence highlights persistent tensions between utilitarian and conservation-oriented perspectives within the hunting community (Díaz-Fernández et al. 2013; Martínez-Jauregui et al. 2017). At the same time, the stronger support among big game hunters suggests an ecological awareness that recognises predators and other non-game species as components of ecosystem balance (Redpath et al. 2013). Incorporating this criterion into certification may therefore help consolidate shared standards of coexistence and ecological stewardship, potentially reducing conflict and strengthening social legitimacy (Fischer et al. 2015; De Bonn et al. 2022).

Criteria associated with social and experiential dimensions played a secondary but non-negligible role. The promotion of local employment and worker training (C6) was significant mainly for small game hunters, reflecting the direct role of gamekeepers in habitat management and stock maintenance in these systems (Arroyo et al. 2012; Delibes-Mateos et al. 2014). Hunter satisfaction (C7), although less frequently perceived as fulfilled, was statistically significant in the models, underscoring that experiential dimensions—such as enjoyment, fairness, and perceived legitimacy—form part of how sustainability is evaluated by hunters (Manning 2011; Vaske and Roemer 2013; Linares et al. 2024b).

### Implications for certification and hunting governance

Taken together, the results suggest that sustainability certification in recreational hunting is more likely to gain acceptance when it aligns with hunters’ self-perceived practices and priorities. Certification appears to function less as a corrective instrument and more as a mechanism for transparency, recognition, and differentiation among hunting estates. This supports interpretations of certification as an information-based governance tool capable of addressing information asymmetries while remaining sensitive to user motivations (Blackman and Rivera 2011; Potts et al. 2014).

More broadly, the Spanish scheme can be situated within discussions of hunting-related certification as a voluntary governance instrument (Child and Wall 2009; Wanger et al. 2017). While these proposals differ in design, geographic context, and governance objectives, a shared challenge concerns ensuring that certification is perceived as legitimate among hunters, a condition that, as our findings confirm, is central to the acceptance of any scheme.

At the same time, the findings indicate that certification should not be interpreted as a guarantee of transformative governance change. The discrete choice experiment captures stated preferences under hypothetical conditions and does not directly measure behavioural change or institutional transformation. Accordingly, the results support modest, context-dependent governance implications rather than strong claims about value shifts. Similar studies in other natural resource contexts suggest that stated preferences can nevertheless signal conditional support for governance arrangements perceived as legitimate and meaningful (Fischer et al. 2015; Castillo-Eguskitza et al. 2019).

From this perspective, discrete choice experiments provide an empirical bridge between individual preferences and collective governance. By analysing trade-offs among sustainability attributes, DCEs reveal how hunters weigh ecological, social, and experiential considerations when evaluating certification, and how legitimacy and trust condition their acceptance (Gupta 2008; Domínguez-Torreiro and Soliño, 2011). These signals are particularly relevant in contexts where governance relies on voluntary uptake rather than enforcement.

Hunting practices are embedded in shared moral frameworks that underpin both collective identity and social legitimacy (von Essen et al. 2015). These frameworks are flexible: hunters balance ethical considerations with achievement-oriented motivations and the practical realities of harvest (Fulton and Manfredo 2004; Brunke and Hunt 2008). Comparable evidence from North America shows that hunters also oppose management practices perceived as harmful to ecosystem integrity, particularly when such practices undermine biodiversity or long-term hunting sustainability (Ghasemi and Kyle 2023). This convergence across contexts reinforces the view that ecological concerns can coexist with achievement-oriented motivations among hunters, while also highlighting the diversity of underlying normative and identity-based pathways. In this sense, certification can be understood as an institutional translation of these community-based ethics into transparent and externally verifiable standards, potentially reinforcing hunting’s social licence in contexts of heightened public scrutiny (Gehman et al. 2017).

Several limitations should be acknowledged. The use of a non-probabilistic snowball sampling strategy limits generalisability and may have favoured hunters more engaged with management issues. As a stated-preference method, the discrete choice experiment captures hypothetical choices rather than observed behaviour, and the cost attribute was not salient for all respondents, constraining willingness-to-pay interpretation. These limitations delimit the scope of inference and highlight the need for complementary behavioural and qualitative research.

Overall, the study contributes to debates on the governance and legitimacy of recreational hunting by empirically examining how hunters evaluate sustainability criteria and certification under different framing conditions. By highlighting both the opportunities and limits of certification as a governance tool, the findings point to the importance of context-aware instruments that balance ecological objectives with social acceptance and experiential values.

## Conclusions

The analysis of hunters’ preferences may have implications for a transformative governance and practice, towards a sustainable system recognising the relevance of recreational hunting and wildlife management as part of the cultural identity of regions. The results highlight that hunters’ support for sustainability certification is conditional and context-dependent, shaped by both ecological awareness and achievement-oriented motivations. Recognising this dual rationale is crucial for designing certification schemes that are not only ecologically sound but also socially credible. While hunters may be reluctant to pay for certification when they perceive that sustainable management has already been achieved, their support for certification as a principle suggests possible indirect effects. By creating reputational incentives for transparency and accountability, certification could encourage landowners to adopt and maintain certified management practices, thus strengthening the social legitimacy of recreational hunting. Certification systems that reward responsible management, strengthen transparency, and respect hunters’ experiential values can promote behavioural change without eroding autonomy or satisfaction. At the same time, integrating education into the ecological consequences of practices such as restocking or predator control could foster shared learning and trust among the actors involved in hunting management. By linking sustainability standards to the lived experience of hunters, certification can become a bridge between conservation goals and the social realities of rural landscapes, contributing to more inclusive and adaptive governance.

## Supplementary Information

Below is the link to the electronic supplementary material.

Supplementary file1 (PDF 564 KB)

## Data Availability

The data supporting the findings of this study are available from the corresponding author upon request.
