# Supplementary material for: Between conservation and achievement: Hunters’ values and sustainability certification as a governance tool in recreational hunting
Source: Ambio. 2026 May 13;55(12):2932–48. doi: 10.1007/s13280-026-02412-z (PMC13731631; doi:10.1007/s13280-026-02412-z)
Supplement: Supplementary file 1 [file 13280_2026_2412_MOESM1_ESM.pdf]

Between conservation and achievement: Hunters' values and sustainability certification as a governance tool in recreational hunting

Olmo Linares[1], Mario Soliño[2], Juan Carranza[1], María Martínez-Jauregui[3]

[1] Unidad de Investigación en Recursos Cinegéticos y Piscícolas (UIRCP); Universidad de Córdoba; Campus de Rabanales Colonia San José, Ctra. Nacional IV – A, Km 396, 14071, Córdoba, (Spain).
[2] Instituto de Investigaciones Marinas (IIM-CSIC). Rúa Eduardo Cabello 6, 36208, Vigo, Spain.
[3] Instituto de Ciencias Forestales (ICIFOR, INIA-CSIC), Ctra. de La Coruña km 7.5, 28040 Madrid (Spain)

Corresponding author e-mail: b02lieso@uco.es

ORCID (Olmo Linares): 0000-0002-9826-7920
ORCID (Mario Soliño): 0000-0001-7065-7348
ORCID (Juan Carranza): 0000-0002-0368-7173
ORCID (María Martínez-Jauregui): 0000-0002-7590-822X

Abstract

Understanding how resource users perceive and negotiate sustainability is essential for designing legitimate conservation governance. This study explores recreational hunters' preferences for sustainability certification in Spain using a discrete choice experiment. Data were collected from 621 hunters representing both big game and small game modalities. Results show that hunters consistently prioritise ecological criteria—such as game population conservation, habitat management, and wild genetics—yet support for certification is conditional on maintaining satisfactory hunting outcomes. Pro-sustainability attitudes thus coexist with achievement-oriented motivations, with conservation valued when it sustains continuity, fairness, and authenticity of the hunting experience. These findings highlight how ecological and experiential rationalities can align rather than conflict. By revealing the conditions under which certification is perceived as acceptable, the study shows how sustainability standards can function as information-based governance tools that enhance legitimacy, trust, and adaptive management in recreational hunting systems.

Key words: Achievement motivation; Discrete choice experiment; Human–wildlife relations; Recreational hunting; Sustainability certification; Transformative governance

Acknowledgements: We thank the regional and national technicians and directors of the "Royal Spanish Hunting Federation" and especially the delegations of Andalusia and Extremadura for their commitment to the study and help in the distribution of the questionnaires.

Author Contribution declaration: All authors contributed to defining the research objectives, discussing the results, and revising the manuscript. The questionnaire was designed collaboratively by MMJ, OL, and MS. MMJ prepared the initial draft of the article, and MS performed the statistical analyses. OL coordinated the fieldwork and led the substantial rewriting, restructuring, and development of the revised manuscript, including the integration of reviewer comments and follow-up of the revision process. JC provided critical revisions and feedback.

Competing Interests: The authors have no competing interests to declare that are relevant to the content of this article.

AUTHOR BIOGRAPHIES

Olmo Linares is a Postdoctoral Researcher at the Wildlife Research Unit (UIRCP), University of Córdoba, Spain. His research focuses on the human dimensions of wildlife management, participatory and inclusive governance processes, and sustainability certification in recreational hunting. He integrates ecological and social approaches to develop governance frameworks that reconcile biodiversity conservation with rural livelihoods. Address: Unidad de Investigación en Recursos Cinegéticos y Piscícolas, Universidad de Córdoba, Campus de Rabanales, Ctra. N-IV A, Km 396, 14071 Córdoba, Spain. e-mail: b02lieso@uco.es

Mario Soliño is a Permanent Researcher at the Instituto de Investigaciones Marinas (IIM-CSIC) in Vigo, Spain. His research focuses on the economic valuation of ecosystem services, particularly non-market goods such as biodiversity conservation. He employs stated preference methods to assess societal values, aiming to inform sustainable management of natural resources, including marine and coastal ecosystems. Address: Instituto de Investigaciones Marinas, CSIC, Rúa Eduardo Cabello 6, 36208 Vigo, Spain. e-mail: msolino@iim.csic.es

Juan Carranza is a Full Professor of Zoology and Director of the Wildlife Research Unit (UIRCP) at the University of Córdoba, Spain. His research focuses on the ecology, behavior, and management of red deer, addressing topics such as sexual selection, mating systems, population dynamics, and habitat management. He has integrated evolutionary and applied approaches to develop sustainable management models for deer populations. Address: Unidad de Investigación en Recursos Cinegéticos y Piscícolas, Universidad de Córdoba, Campus de Rabanales, Ctra. N-IV A, Km 396, 14071 Córdoba, Spain. e-mail: juan.carranza@uco.es

María Martínez-Jauregui is a Permanent Researcher at the Forest Research Centre of the Instituto Nacional de Investigación y Tecnología Agraria y Alimentaria (INIA-CSIC) in Madrid, Spain. Her research interests include wildlife ecology, conservation, and management, with a focus on integrating ecological and socioeconomic perspectives to enhance the effectiveness of conservation strategies in the context of global change. Address: Centro de Investigación Forestal (INIA-CSIC), Carretera La Coruña, 7.5 Km, 28040 Madrid, Spain. e-mail: martinezmari@gmail.com

Questionnaire

This survey is part of a study promoted by the Chair of Game and Fish Resources on the certification of sustainable game management in hunting grounds. We would like to know your opinion on this subject and therefore we ask for your collaboration by answering a series of questions related both to your hunting activity during the last hunting season and your preferences regarding different issues related to the certification of sustainable game management.

Step1

1.- Did you hunt in the last hunting season in Andalusia?

□ Yes; □ No

Step2

2.- In the last season, what did you practice most frequently in Andalusia:

□ Big game hunting in a closed, social hunting ground
□ Big game hunting in a closed, private hunting ground
□ Big game hunting in an open, social hunting ground
□ Big game hunting in an open, private hunting ground
□ Small game hunting in a social hunting ground
□ Small game hunting in a private hunting ground

Step3

3. How far is the hunting ground where you hunted most frequently during the last season from your primary residence?

______________ km from your residence to the hunting ground.

4.- From now on, we will refer to the hunting ground where you hunted most frequently in Andalusia in the past season and we will call it your "usual hunting ground". Which of the following issues was decisive for you to have attended your usual hunting ground? (dropdown, one option)

□ Because the hunting ground is well managed
□ Because my association or society manages it
□ Because I was invited
□ It is close to my place of residence
□ The probability of bagging game is very high
□ Because of the possibility of bagging medal-worthy trophies
□ Because of the natural environment
□ Out of tradition
□ Because this is where my friends or family go
□ Value for money
□ Other (please specify: ______________________)

Step4

5.- Please indicate the number of hunting days for each modality you practiced in your "usual hunting ground" in the last season.

Big game	Small game
__ days of Stalking (*Rececho*)	__ days of Driven Partridge Shoot (*Ojeo de perdiz*)
__ days of Ambush (*Aguardo*)	__ days of Decoy Hunting (*Reclamo*)
__ days of Big Driven Hunt (*Montería/Gancho*)	__ days of Walk-Up Hunting (*Al salto*)
__ days of Beat (*Batida*)	__ days of Walked-Up Hunting (*En mano*)
__ days of Selective Culling (*Selectiva*)	__ days Other >> specify: __
__ days Other >>specify: __	

7. How much did you pay to hunt in your usual hunting ground in the past season (add the total cost for being able to hunt all days in the usual ground and do not include ammunition, clothing, food, transport,…)?

_______________ euros

Step6

8.- Are you aware of any initiative to certify SUSTAINABLE GAME MANAGEMENT in Spanish hunting grounds?

□ No

□ Yes, please specify: ______

Step7

9.- SUSTAINABLE GAME MANAGEMENT is the management of game species and their habitats in a way and at a rate that does not lead to a long-term decline in biodiversity or hinder its restoration. Sustainable game management must meet the needs and aspirations of present and future generations.

Previous experiences identify 7 necessary criteria to CERTIFY, by an independent entity, SUSTAINABLE GAME MANAGEMENT. This certification would mean that the management of the hunting ground is based on the sustainable use of resources and that it offers hunting that contributes to the ecosystem's balance.

We ask you to give us your opinion on each of the 7 CRITERIA and whether you believe your usual hunting ground meets them.

Step8

Criterion 1

Criterio 1

Conservation of game population

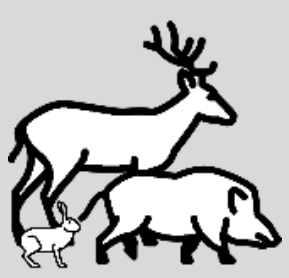

Respecting this criterion implies:

- For big game: Maintaining a balance of ages and sexes and avoiding high densities harmful to the species and the ecosystem.
- For small game: Ensuring a minimum density of wild-born individuals that allows hunting without compromising the population.

Please rate the importance of this criterion for achieving sustainable game management from 1 to 10 (where 1: not important and 10: very important): _____

Please indicate if you believe your usual hunting ground is managed respecting this criterion:
□ Yes, I believe it respects it
□ No, I believe it does not respect it
□ I don't know, I'm not sure

Step9
Criterion 2

**Criterion 2**<br>**Promotion of wild and native genetics**	
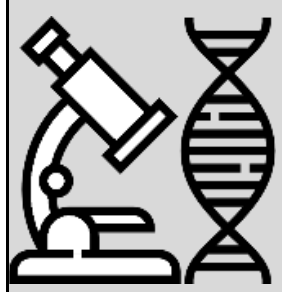	Respecting this criterion implies:<br>• Maintaining the pure-native genetics of the hunted populations.<br>• Maintaining wild genetics by avoiding practices that alter natural selection processes.<br>• Not introducing farm-reared animals as a system to ensure hunting.

Please rate the importance of this criterion for achieving sustainable game management from 1 to 10 (where 1: not important and 10: very important): _____

Please indicate if you believe your usual hunting ground is managed respecting this criterion:
□ Yes, I believe it respects it
□ No, I believe it does not respect it
□ I don't know, I'm not sure

Step10
Criterion 3

**Criterion 3**<br>**Avoid commercial hunting of exotic species**	
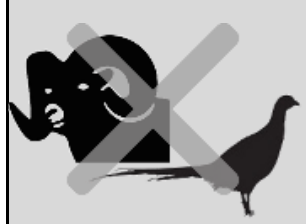	Respecting this criterion implies:<br>• Eliminating the commercial hunting of exotic species such as aoudad, mouflon, pheasant...<br>• Promoting only game species native to the area.

Please rate the importance of this criterion for achieving sustainable game management from 1 to 10 (where 1: not important and 10: very important): _____

Please indicate if you believe your usual hunting ground is managed respecting this criterion:
□ Yes, I believe it respects it
□ No, I believe it does not respect it
□ I don't know, I'm not sure

Step11
Criterion 4

**Criterion 4**<br>**Maintain or improve non-game wildlife**	
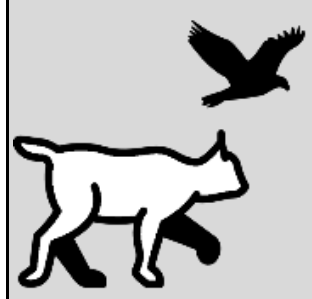	Respetar este criterio implica:<br>• Knowing what fauna inhabits the hunting ground and caring for the populations of wild species that are not hunted (small birds, raptors, lynxes, reptiles…).

Please rate the importance of this criterion for achieving sustainable game management from 1 to 10 (where 1: not important and 10: very important): _____

Please indicate if you believe your usual hunting ground is managed respecting this criterion:
□ Yes, I believe it respects it
□ No, I believe it does not respect it
□ I don't know, I'm not sure

Step12
Criterion 5

**Criterion 5**<br>**Manage and conserve habitats**	
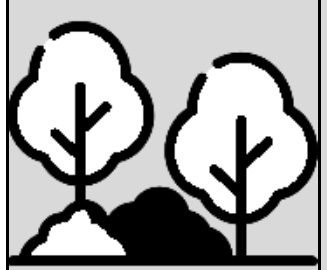	Respecting this criterion implies:<br>• Ensuring the renewal of trees and woody plants.<br>• Avoiding soil compaction and erosion.<br>• Conserving hedgerows, borders, and patches of vegetation.<br>• Promoting adequate use of phytosanitary products.<br>• Ensuring agricultural tasks respect the breeding seasons of species.

Please rate the importance of this criterion for achieving sustainable game management from 1 to 10 (where 1: not important and 10: very important): _____

Please indicate if you believe your usual hunting ground is managed respecting this criterion:
□ Yes, I believe it respects it
□ No, I believe it does not respect it
□ I don't know, I'm not sure

Step13
Criterion 6

**Criterion 6**<br>**Promote local employment and worker training**	
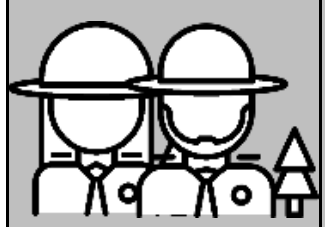	Respecting this criterion implies:<br>• The staff of the hunting ground are properly trained and have the necessary means.<br>• Hiring personnel from the area where the hunting ground is located is favored.

Please rate the importance of this criterion for achieving sustainable game management from 1 to 10 (where 1: not important and 10: very important): _____

Please indicate if you believe your usual hunting ground is managed respecting this criterion:
□ Yes, I believe it respects it
□ No, I believe it does not respect it
□ I don't know, I'm not sure

Step14
Criterion 7

**Criterion 7**<br>**Increase customer satisfaction**	
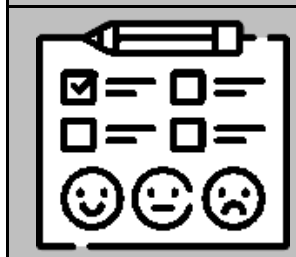	Respecting this criterion implies :<br>• Asking and recording the hunter's satisfaction at the end of the hunt.<br>• The hunter has mechanisms to claim if what was offered to them is not fulfilled.

Please rate the importance of this criterion for achieving sustainable game management from 1 to 10 (where 1: not important and 10: very important): _____

Please indicate if you believe your usual hunting ground is managed respecting this criterion:
□ Yes, I believe it respects it
□ No, I believe it does not respect it
□ I don't know, I'm not sure

Step15

25. Taking into account that last season you paid [information 2] for all the days in your usual hunting ground, if your usual hunting ground had been certified for the 7 criteria of sustainable game management, would you be willing to pay [information 3] for those same days in your usual hunting ground?

PAYMENT CARD WITH 11 AMOUNTS
[CALCULATE 0%-5%-10%-15%-…-50% ON [information 2]

Step17

27. Any certified hunting ground in Andalusia could mean an increase in the price to hunt there, which will depend on the criteria that are certified. Below, we propose several hypothetical hunting grounds where some criteria are certified and others are not. We ask that in each card you look at the certification status of these criteria and indicate whether you would be willing to assume the additional cost to hunt in that ground. Remember that paying more for any of these alternatives means giving up other consumption or saving options.

Certified Criteria	Certificate A	Certificate B	Without Certificate
Cr.1.-Conservation of game populations		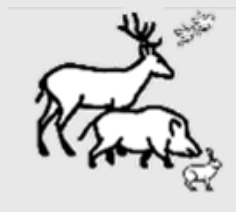	
Cr.2.- Promotion of wild and native genetics	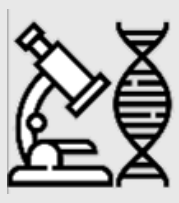		
Cr.3.- Avoid commercial hunting of exotic species		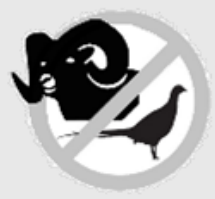	
Cr.4.- Maintain or improve non-game wildlife		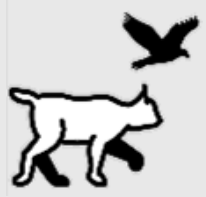	
Cr.5.- Manage and conserve habitats	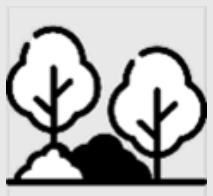		
Cr.6.- Promote local employment and worker training		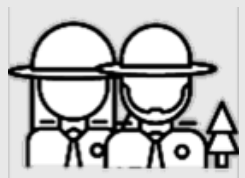	
Cr.7.- Increase hunter satisfaction		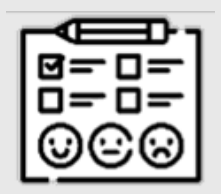	
Cost of certification	30 €	60 €	0 €
Tick your preferred alternative	☐	☐	☐

Step18

28. [Only if "None" was always chosen] Why would you not be willing to pay more to attend a hunting ground that meets certain criteria of SUSTAINABLE GAME MANAGEMENT? (You can mark several)

□ Because I cannot afford to pay more

□ Because I am not interested in sustainable game management certification

□ Because I did not understand what they were asking
□ Other (please specify: ______________________________________)

Step19
29.- Gender: □ Male □ Female
30. Year of birth: __ __ __ __
31. In which municipality is your primary residence? ________

32. Indicate the number of people in your household unit (household unit is understood as all people who depend directly on your family's economy):
YOURSELF: 1
Spouse or partner: _____
Children: _____
Father and/or mother or other relatives: _____

33. Please indicate the range where your net monthly income (after taxes) falls, both your own exclusively and that of your family.

Net Monthly Income	Your Own	Family's
No income	□	□
Less than 600 euros	□	□
Between 600 euros and 1,200 euros	□	□
Between 1,201 euros and 1,800 euros	□	□
Between 1,801 euros and 2,400 euros	□	□
Between 2,401 euros and 3,000 euros	□	□
Between 3,001 euros and 3,600 euros	□	□
Between 3,601 euros and 4,200 euros	□	□
Above 4,200 euros	□	□

□ Many people do not answer this question and it is really important for our analysis. We just want to remind you that the information provided is completely confidential and once received, it cannot be associated with your person. However, you are free not to answer. Please check this box if you do not want to give us information about your income or that of your family.

Step20
34. The survey is almost finished. Any additional comments you would like to make about game management certification are welcome:
□ I have no comments
□ Comment: ____

36. THANK YOU VERY MUCH FOR YOUR COLLABORATION

CLICK "FINISH" TO COMPLETE THE SURVEY >>>>>>>>>>>> □ FINISH

Annex 1. Choice cards (1: the criteria is accomplished; 0: the criteria is not accomplished)

Choice card # 1	Certificate A	Certificate B	Without certificate
C1	0	1	0
C2	1	0	0
C3	0	1	0
C4	0	1	0
C5	1	0	0
C6	0	1	0
C7	0	1	0
Cost (% increasing)	10	15	0

Choice card # 2	Certificate A	Certificate B	Without certificate
C1	1	0	0
C2	0	1	0
C3	1	0	0
C4	0	1	0
C5	0	1	0
C6	1	0	0
C7	0	1	0
Cost (% increasing)	5	10	0

Choice card # 3	Certificate A	Certificate B	Without certificate
C1	0	1	0
C2	1	0	0
C3	1	0	0
C4	1	0	0
C5	0	1	0
C6	1	0	0
C7	0	1	0
Cost (% increasing)	10	15	0

Choice card # 4	Certificate A	Certificate B	Without certificate
C1	1	0	0
C2	1	0	0
C3	0	1	0
C4	1	0	0
C5	0	1	0
C6	0	1	0
C7	1	0	0
Cost (% increasing)	5	10	0

Choice card # 5	Certificate A	Certificate B	Without certificate
C1	0	1	0
C2	0	1	0
C3	1	0	0
C4	1	0	0
C5	1	0	0
C6	0	1	0
C7	1	0	0
Cost (% increasing)	5	10	0

Choice card # 6	Certificate A	Certificate B	Without certificate
C1	1	0	0
C2	0	1	0
C3	0	1	0
C4	0	1	0
C5	1	0	0
C6	1	0	0
C7	1	0	0
Cost (% increasing)	10	15	0

Choice card # 7	Certificate A	Certificate B	Without certificate
C1	1	0	0
C2	0	1	0
C3	0	1	0
C4	1	0	0
C5	0	1	0
C6	0	1	0
C7	0	1	0
Cost (% increasing)	20	5	0

Choice card # 8	Certificate A	Certificate B	Without certificate
C1	1	0	0
C2	1	0	0
C3	1	0	0
C4	0	1	0
C5	1	0	0
C6	0	1	0
C7	0	1	0
Cost (% increasing)	15	20	0

Choice card # 9	Certificate A	Certificate B	Without certificate
C1	0	1	0
C2	0	1	0
C3	0	1	0
C4	1	0	0
C5	1	0	0
C6	1	0	0
C7	0	1	0
Cost (% increasing)	15	20	0

Choice card # 10	Certificate A	Certificate B	Without certificate
C1	0	1	0
C2	0	1	0
C3	1	0	0
C4	0	1	0
C5	0	1	0
C6	0	1	0
C7	1	0	0
Cost (% increasing)	20	5	0

Choice card # 11	Certificate A	Certificate B	Without certificate
C1	0	1	0
C2	1	0	0
C3	0	1	0
C4	0	1	0
C5	0	1	0
C6	1	0	0
C7	1	0	0
Cost (% increasing)	15	20	0

Choice card # 12	Certificate A	Certificate B	Without certificate
C1	1	0	0
C2	1	0	0
C3	1	0	0
C4	1	0	0
C5	1	0	0
C6	1	0	0
C7	1	0	0
Cost (% increasing)	20	5	0
